# c-Myb regulates tumorigenic potential of embryonal rhabdomyosarcoma cells

**DOI:** 10.1038/s41598-019-42684-y

**Published:** 2019-04-19

**Authors:** Petr Kaspar, Jan Prochazka, Michaela Efenberkova, Attila Juhasz, Vendula Novosadova, Radislav Sedlacek

**Affiliations:** 1 Laboratory of Transgenic Models of Diseases, Institute of Molecular Genetics of the Czech Academy of Sciences, Prague, 14220 Czech Republic; 2 Microscopy Centre – LM and EM, Institute of Molecular Genetics of the Czech Academy of Sciences, Prague, 14220 Czech Republic; 3 Czech Centre for Phenogenomics Institute of Molecular Genetics of the Czech Academy of Sciences, Prague, 14220 Czech Republic

**Keywords:** Oncogenes, Sarcoma, Cell growth

## Abstract

Rhabdomyosarcomas (RMS) are a heterogeneous group of mesodermal tumors, the most common sub-types are embryonal (eRMS) and alveolar (aRMS) rhabdomyosarcoma. Immunohistochemical analysis revealed c-Myb expression in both eRMS and aRMS. c-Myb has been reported to be often associated with malignant human cancers. We therefore investigated the c-Myb role in RMS using cellular models of RMS. Specific suppression of c-Myb by a lentiviral vector expressing doxycycline (Dox)-inducible c-Myb shRNA inhibited proliferation, colony formation, and migration of the eRMS cell line (RD), but not of the aRMS cell line (RH30). Upon c-Myb knockdown in eRMS cells, cells accumulated in G0/G1 phase, the invasive behaviour of cells was repressed, and elevated levels of myosin heavy chain, marker of muscle differentiation, was detected. Next, we used an RD-based xenograft model to investigate the role of c-Myb in eRMS tumorigenesis *in vivo*. We found that Dox administration did not result in efficient suppression of c-Myb in growing tumors. However, when c-Myb-deficient RD cells were implanted into SCID mice, we observed inefficient tumor grafting and attenuation of tumor growth during the initial stages of tumor expansion. The presented study suggests that c-Myb could be a therapeutic target in embryonal rhabdomyosarcoma assuming that its expression is ablated.

## Introduction

Rhabdomyosarcoma (RMS) is the most common pediatric soft tissue sarcoma^1^. These sarcomas represent a heterogeneous group of malignancies that express myogenic regulatory transcription factors such as MyoD, myogenin^2^ and myf5^3,4^ and exhibit defective skeletal muscle differentiation^5^. Myogenin has often been used as specific marker to diagnose RMS^6,7^. RMS are divided into two major histologic subtypes: embryonal (eRMS) and alveolar (aRMS). aRMS is a less frequent and more aggressive disease, predominantly characterized (85%) by the t(2;13) or t(1;13) chromosomal translocation resulting in the fusion of the DNA binding domain of the PAX3 and PAX7 genes to the transactivation domain of the FOXO1 gene^8^. The expression of PAX3/7-FOXO1 fusion proteins correlates with poor clinical outcome and these proteins are considered to be the main factor driving aRMS tumorigenesis^9^. eRMS, in contrast, is genetically more heterogeneous; the loss of heterozygosity of 11p15.5, p53 pathway disruption and RAS activation is often observed in eRMS tumors^10^.

c-Myb is the transcription factor essential for normal adult hematopoiesis^11^. c-Myb plays a role hematopoietic commitment of progenitor cells^12^ and maintenance of proliferative progenitor-cell phenotype; its downregulation is necessary for progenitor cell differentiation^13,14^. c-Myb also regulates progenitor cells in the colonic crypts^15^ and a neurogenic region in the adult brain^16^ and was described as anti-myogenic gene^17^. Due to its role in lineage commitment and regulation of other transcription factors, c-Myb was described as a master regulator^18–21^ able to function as a pioneer transcription factor^22^. It was also shown that c-Myb-specific binding sites vary depending on cell type, reflecting its roles in lineage determination, proliferation and differentiation^23^. The *c-myb* gene is frequently rearranged in many human malignancies; in some cancers amplification of the *myb* gene occurred, resulting in increased c-Myb expression^13,24^.

We have shown that c-Myb is involved in the biology of satellite cells and myoblasts regulating the differentiation program of myogenic progenitor cells^25^. Moreover, we revealed c-Myb expression in both eRMS and aRMS tumor specimens as well as in representative rhabdomyosarcoma cell lines: RD and RH30^26^. Given the c-Myb positivity in RMS we decided to elucidate whether the oncogenic activity of c-Myb is also applied in RMS tumorigenesis.

## Results

### c-Myb suppression inhibits proliferation of eRMS but not aRMS cells

To investigate whether c-Myb plays a role in RMS tumorigenesis, we assessed the effects of c-Myb suppression in embryonal (RD) and alveolar (RH30) RMS cell lines^27^. Since c-Myb has been shown to regulate proliferation in many cell types, we analysed the effect of c-Myb knockdown on the proliferation (measured by ATP assay) of these RMS cell lines. Both cell lines were transduced with the Dox-inducible, GFP-expressing pLVTSH-Myb shRNA lentiviral vector (shMYB), or empty pLVTSH (Empty)^28^ that was used together with the parental cell line as a control.

**Figure 1:**
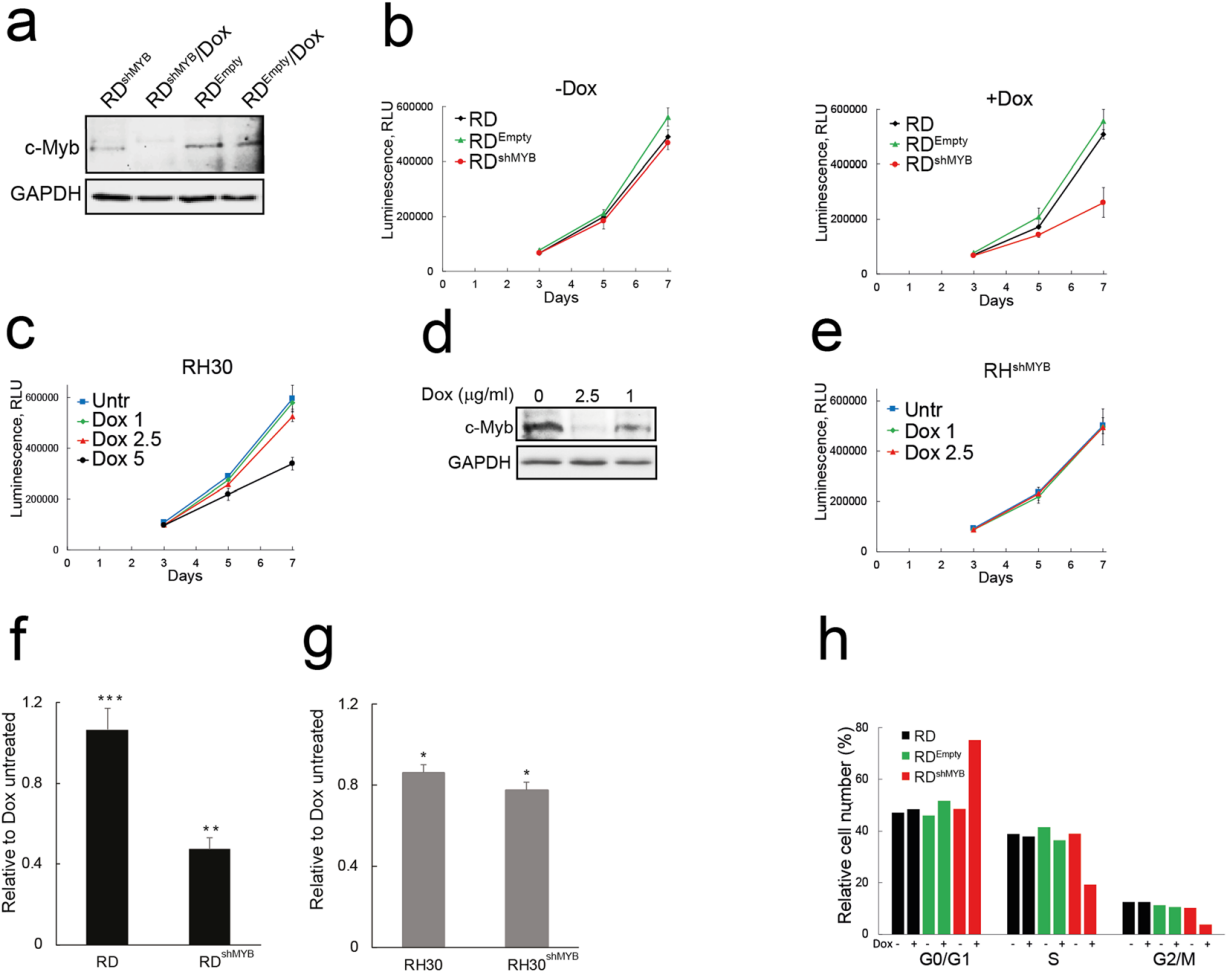
c-Myb suppression leads to inhibition of proliferation of eRMS cell line RD but not aRMS cell line RH30. (**a**) Western blot shows c-Myb expression in RD cells lentivirally transduced with a Dox - inducible c-Myb shRNA (RD^shMYB^) vector or empty vector (RD^Empty^) 48 hours after Dox induction (5 μg/ml). GAPDH served as a loading control. The original full-length blots are presented in Supplementary Fig. 1. (**b**) The proliferation of parental RD and lentivirally transduced RD^shMYB^ and RD^Empty^ cells as measured by ATP assay. Cells were grown with (+Dox) at 5 μg/ml or without Dox (−Dox). (**c**) The effect of increasing Dox concentration on the proliferation of RH30 cells as measured by ATP assay. The Dox concentration used was: 1 μg/ml (Dox 1), 2.5 μg/ml (Dox 2.5), and 5 μg/ml (Dox 5) and compared with untreated cells (Untr). (**d**) Western blot shows c-Myb expression in RH30 cell lentivirally transduced with a Dox-inducible c-Myb shRNA (RH30^shMYB^) vector and treated with Dox at 1 and 2.5 μg /ml. GAPDH served as a loading control. The original full-length blots are presented in Supplementary Fig. 1. (**e**) Proliferation of RH30^shMYB^ cells as measured by ATP assay after treatment with Dox at 1 μg/ml (Dox 1) and 2.5 μg/ml (Dox 2.5). Dox-untreated cells (Untr) served as a control. Comparison of the effect of c-Myb silencing on RD (**f**) and RH30 (**g**) cell line proliferation after six days of treatment with or without Dox as measured by crystal violet staining. RD cells were treated with Dox at 5 μg/ml Dox, RH30 cells with Dox at 2.5 μg/ml. (**h**) Knockdown of c-Myb in RD blocks cell cycle progression. Cells were grown with or without Dox, as indicated, (Dox at 5 μg/ml) for four days and analysed by propidium staining and flow cytometry.

In the RD cell line, Dox induction (5 μg/ml)^28^ of Myb shRNA abolished c-Myb expression, but the c-Myb levels were not affected in cell transduced with empty pLVTSH (Fig. 1a). Dox-induced knockdown of c-Myb resulted in inhibition of proliferation (Fig. 1b); control RD cells were not affected by Dox.

However, RH30 cells were shown to be sensitive to Dox; Dox at 5 μg/ml concentration caused inhibition of proliferation not only of RH^shMYB^ cells, but also of both parental RH30 and empty pLVTSH-transduced control cells RH^Empty^ (Supplementary Fig. 2). While Dox at 5 μg/ml reduced the proliferation rate of parental RH30 cells, starting from Dox 2.5 μg/ml the inhibition was almost extinguished (Fig. 1c). Dox at 2.5 μg/ml also induced c-Myb knockdown as confirmed by western blotting (Fig. 1d), but c-Myb suppression by Dox induction (2.5 μg/ml) did not result in inhibiting proliferation of RH30 as measured by ATP assay (Fig. 1e). Crystal violet staining of cells (Fig. 1f) again showed that the effect of c-Myb suppression on the proliferation of RD cell was profound; knockdown of c-Myb in RD reduced cell numbers after six days of treatment to less than the half compared to Dox untreated cells (normalized to 1). For RH30 we detected combination of slight inhibition of proliferation caused by Dox itself and the c-Myb knockdown (Fig. 1g). Thus, the effect of c-Myb suppression on proliferation of RH30 was negligible after six days of Dox treatment. The downregulation of c-Myb expression detected using western blotting was also verified on mRNA levels (Supplementary Fig. 3)

Our results indicate that c-Myb plays dichotomous roles in proliferation of RMS cell lines, either supporting proliferation of RD cells or exerting no effect on the proliferation of RH30 cells.

### c-Myb-deficient RD cells are accumulated at G0/G1 and reduced at G2/M

Next, we investigated whether the inhibition of proliferation is a result of block in cell cycle progression. Analysis of the DNA content by flow cytometry revealed (Fig. 1h) that RD cells with suppressed c-Myb accumulated after four days of culture in G0/G1, which was consistent with previous reports showing that c-Myb plays a role in regulating G1/S cell cycle transition^29^, and the cell number in G2/M was reduced as well, which is in agreement with c-Myb involvement in G2/M cell cycle progression^30^. In fact, similar inhibition of the cell cycle progression was detected as early as two days after inducing c-Myb suppression (Supplementary Fig. 4).

### c-Myb suppression inhibits colony formation and migration of RD cells but not of RH30 cells

**Figure 2:**
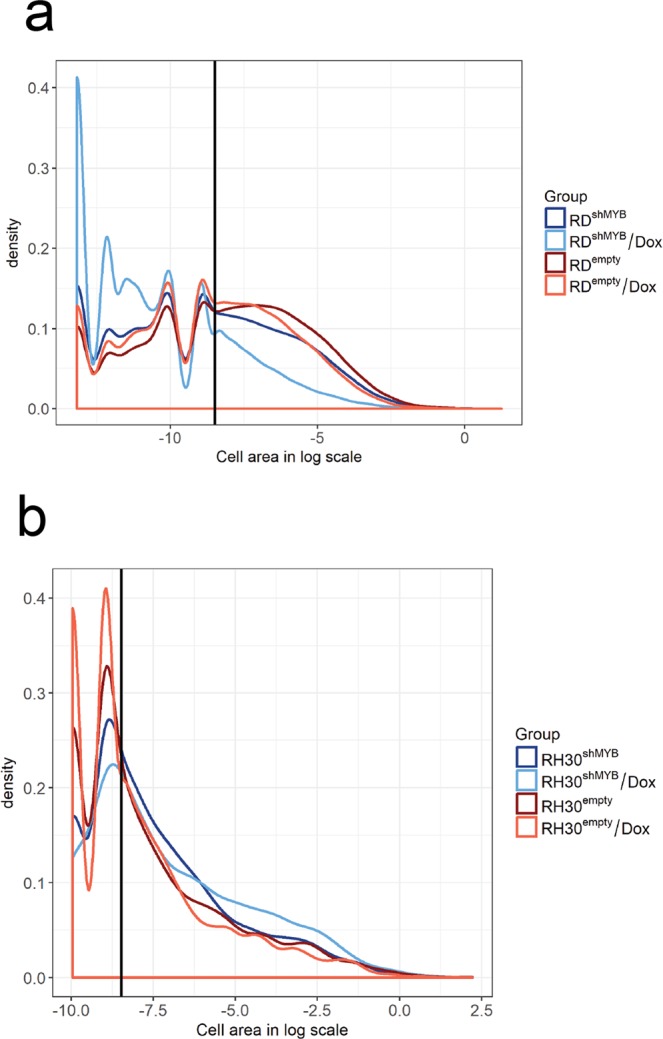
c-Myb suppression inhibits colony formation of eRMS cell line RD but not aRMS cell line RH30. (**a**) RD or (**b**) RH30 cells transduced with a Dox-inducible c-Myb shRNA (RD^shMYB^or RH30^shMYB^) vector or empty vector (RD^Empty^or RH30^Empty^) were cultured with or without Dox (5 μg/ml for RD cells and 2.5 μg/ml for RH30 cells) in 0.35% agar for 21 days. Formed colonies were fixed, stained, the size of colonies was determined, and density plot was created from the log cell area. It is shown that cells form colonies of various sizes: small colonies with a countable number of cells (peaks) are divided from larger colonies formed by an uncountable number of cells by a black line. Higher density shows more cells within the same area.

A well-known characteristic of transformed cells is their ability to grow independently of a solid surface. We therefore investigated whether the anchorage-independent growth ability of RMS cells was affected by c-Myb silencing. We performed a soft agar colony formation assay using both RMS cell lines and demonstrated (Fig. 2a) that c-Myb knockdown strongly inhibited the capacity of RD cells to form large colonies, and accordingly, c-Myb-deficient RD single cells and colonies made of two and three c-Myb-deficient cells were much more abundant compared to colonies made of c-Myb expressing RD cells. However, the formation of large colonies made of RH30 cells was not inhibited by c-Myb suppression (Fig. 2b). These results indicate that c-Myb levels correlate with the transformed cell phenotype of anchorage-independent growth of RD cells.

**Figure 3:**
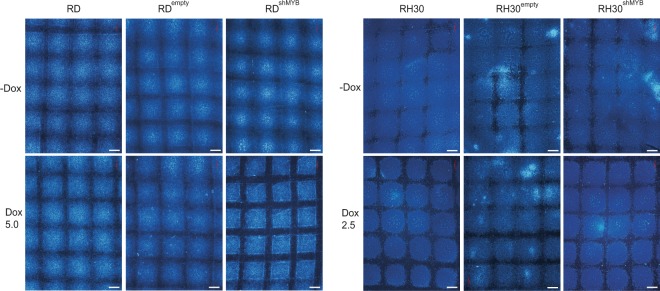
c-Myb suppression inhibits migration of eRMS cell line RD but not of aRMS cell line RH30. Scratch wound healing assay. RD or RH30 cells were transduced with a Dox-inducible c-Myb shRNA (RD^shMYB^or RH30^shMYB^) vector or empty vector (RD^Empty^or RH30^Empty^), cultured with Dox (2.5 μg/ml for RH30 and 5 μg/ml for RD) or without Dox, and subjected to the wound healing assay. Wounds were created by a comb. Wounded cells cultured with or without Dox were incubated for an additional 72 hours and analysed. Scale bars: 1 mm.

The role of c-Myb in stimulating cell migration and invasion has also been reported by several authors. We showed that c-Myb is involved in the migration of C2C12 myoblasts^26^. As cell migration and invasion are hallmarks of metastatic cancers, we examined the migration and invasion of RMS cell lines after inhibition of c-Myb expression. To study the cell migration we performed scratch wound healing experiments using both RD and RH30 cell lines (Fig. 3). In contrast to control RD cells that after 72 hours almost completely filled the wounded area, RD cells with silenced c-Myb did not migrate into the wound, suggesting that c-Myb plays a role in the migration of RD cells. RH30 cells with silenced c-Myb migrated into the wound at the same rate as control RH30 cells, proposing that c-Myb is not involved in RH30 cell migration (small negative effect of Dox at 2.5 μg/ml on the migration was apparent as control RH30 cells treated with Dox moved more slowly than untreated cells).

**Figure 4:**
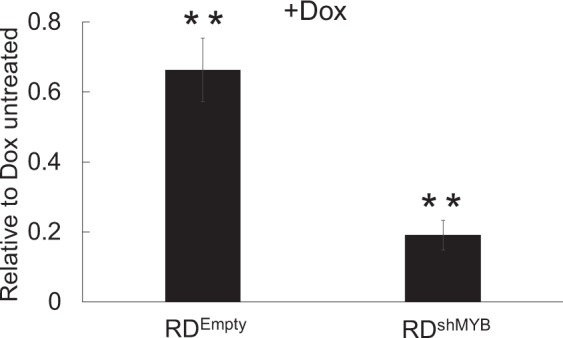
The invasive behavior of eRMS cells is reduced by c-Myb suppression. Transwell migration assay. For the invasive assay, RD cells transduced with a Dox-inducible c-Myb shRNA (RD^shMYB^) vector or empty vector (RD^Empty^) were Dox pretreated or untreated. Then the cells were seeded on the transwell chambers precoated with ECM Matrix gel solution, and the number of cells on the lower side of the insert was counted after 48 h incubation.

To examine the invasion behaviour of c-Myb-deficient RD cells we performed a transwell migration assay using a Matrigel-coated membrane (Fig. 4). We showed that the passage of cells across the Matrigel membrane was dependent on c-Myb, as the cell number of c-Myb-deficient RD^shMYB^ cells was reduced to one fifth compared to Dox untreated RD^shMYB^ cells (normalized to 1). However, we also identified a c-Myb nonspecific minor inhibitory effect of Dox as the number of control RD^Empty^ cells that passed the membrane was slightly reduced when they were treated with Dox compared to RD^Empty^ without Dox.

**Figure 5:**
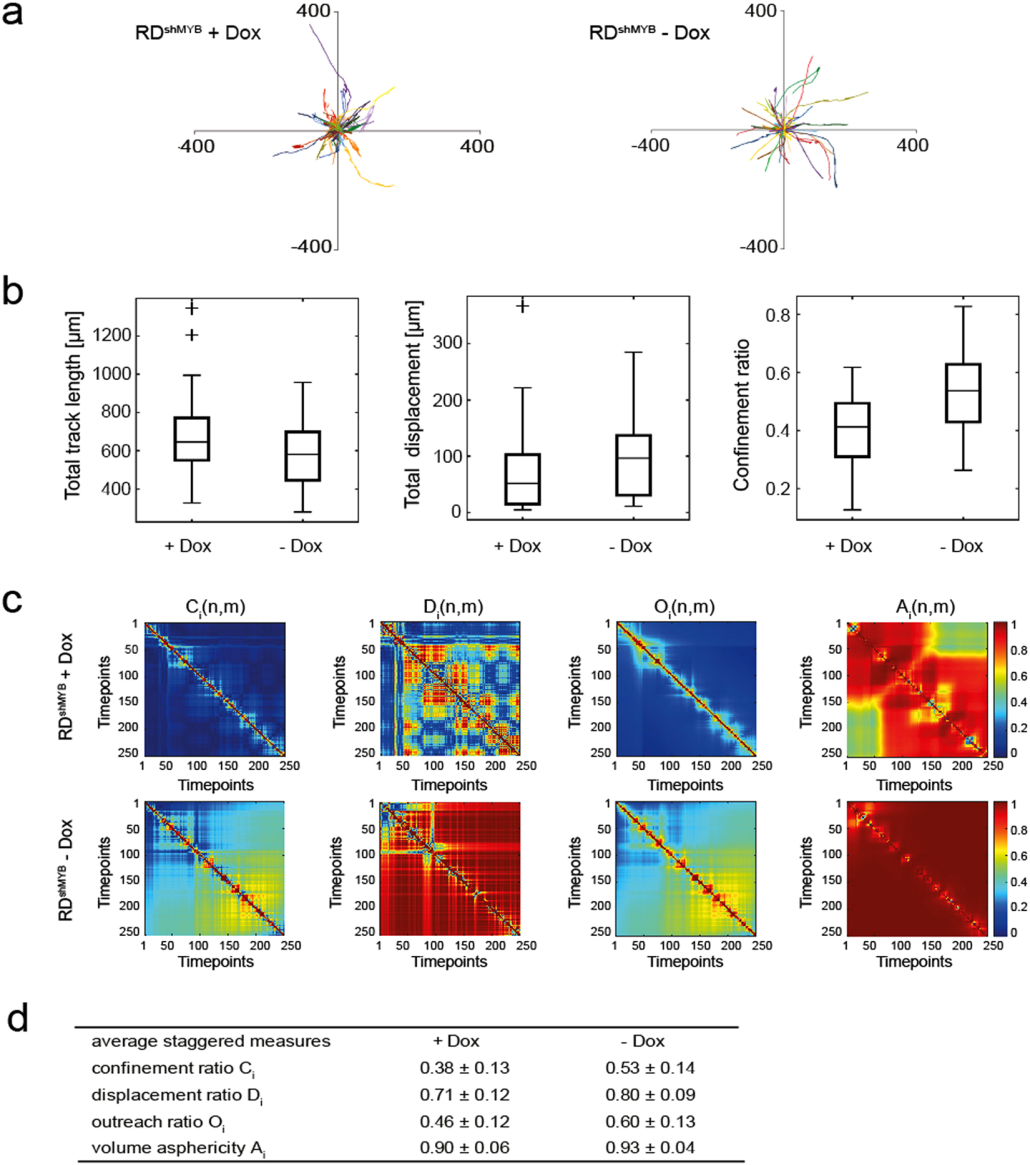
Analyses of cell track data for RD^shMYB^ cells with and without Dox. (**a**) Plot of individual RD^shMYB^ cell tracks after alignment of starting positions. Number of cell tracks *n*_+*Dox*_ = 34, *n*_*−Dox*_ = 30. (**b**) Boxplots showing total track length, total displacement and staggered confinement ratio of cells with and without Dox, respectively. The total track length measure showed no significant difference, while both total track displacement and staggered confinement ratio *(in table D)* were significantly different (*p* < *0.05, p* < *0.01 respectively)*. Crosses denote outliers. (**c**) Heat maps of the staggered confinement ratio, staggered displacement ratio, staggered representative cells with individual values close to the average of staggered measures *(in table D)*. One timepoint corresponds to 2 minutes. (**d**) Average values with standard deviations of all average staggered measures for cells with and without Dox. Wilcoxon rank-sum test was used to compare the average staggered measures of +Dox and –Dox cells. All measures for the two cell types were significantly different (*p* < *0.01*).

Since the scratch wound healing assay and transwell migration assay are indirect observations of cell migration, we performed a time-lapse microscopy migration study of single RD cells. RD^shMYB^ and parental RD cells were co-cultured with or without Dox in one dish to ensure the same culture conditions. The imaging was performed for 48 h (Supplementary Video). To analyse the migratory behaviour of RD cells at a single-cell resolution, we followed at least 30 cells from each experimental group and analysed the individual cell migration characteristics. Individual trajectories (Fig. 5a) of c-Myb-deficient RD cells showed a less uniform behaviour and were in most cases more restrained when compared to the same cells untreated. Surprisingly, we did not observe any significant difference in the total track length of migrating c-Myb-deficient RD cells and the same cells without Dox treatment, but interestingly, the total displacement and confinement ratio was reduced (Fig. 5b), suggesting that c-Myb suppression had no effect on the cell mobility in general, but was more involved in the invasive behaviour during migration. To better understand how the cell migration is affected by c-Myb suppression at a single-cell level, we performed detailed migration analysis according to^31^ using the measures: confinement ratio – referring to how straight the cell migration is between two timepoints, displacement ratio – corresponding to the action radius of the track between two timepoints, outreach ratio – describing the maximal displacement length that is realized within the track segment, and volume asphericity – a characteristic that describes the shape of the track volume, its ellipticity. A representative cell track with all values of staggered measures close to the average value (Fig. 5d) was selected from both populations and analysed. The resulting staggered characteristics were represented as 2D heat maps (Fig. 5c). A c-Myb-deficient cell exhibited a strongly confined type of motion (many dark blue pixels corresponding to low values close to 0 in confinement ratio C_i_, displacement ratio D_i_ and outreach ratio O_i_, and large areas in volume asphericity A_i_ with values different from 1), in contrast to the cells with c-Myb not suppressed that showed active movement with more straight cell tracks (only few pixels with low values in C_i_, D_i_ and O_i_ heat maps and only small areas in A_i_ with values different from 1). Analysis of migration of parental RD showed that the cells were evenly distributed and their trajectories did not differ no matter whether they were treated with Dox or not (Supplementary Fig. 5), indicating that Dox per se did not influence the migration.

Taken together, data from the scratch wound healing assay, transwell assay, and single-cell migration analysis clearly indicate that c-Myb supports invasive migratory behaviour of RD cells.

### c-Myb suppression supports myogenic differentiation of RD cells

**Figure 6:**
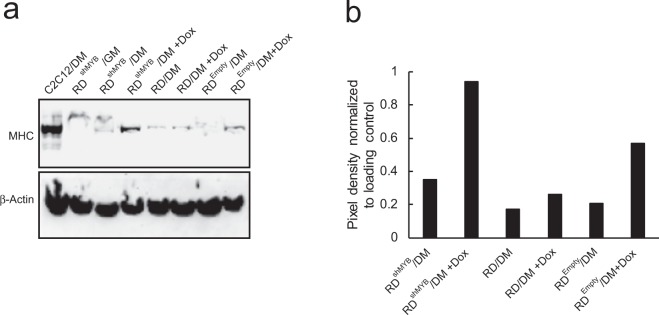
c-Myb suppression promotes myogenic differentiation of eRMS cells. (**a**) Western blotting analysis. RD cells transduced with a Dox-inducible c-Myb shRNA (RD^shMYB^) vector or empty vector (RD^Empty^), parental RD cells, or C2C12 myoblasts were cultured with or without Dox, induced to differentiate by incubation in DM for 72 hours, and immunostained for myosin heavy chain (MHC). GAPDH served as a loading control. The original full-length blots are presented in Supplementary Fig. 1. (**b**) Quantification of MHC specific band in differentiating RD cells.

Since RMS displays defective skeletal muscle differentiation, the restoration of myogenic differentiation could inhibit tumorigenicity. We have shown that skeletal muscle differentiation was blocked by constitutively expressed c-Myb^25^; moreover, RMS cell lines express c-Myb^26^. Therefore, we asked ourselves whether the myogenic program could be restored or enhanced in the absence of c-Myb. We performed differentiation experiments and found that RD cells with c-Myb downregulated did not exhibit fusion of individual cells (a typical feature of skeletal myogenesis seen in differentiating C2C12 cells) and were morphologically similar to untreated cells (Supplementary Fig. 6). However, western blotting for myosin heavy chain (MHC), marker of myogenesis, indicated higher expression of MHC in c-Myb-deficient RD cells (Fig. 6).

We can conclude that the c-Myb deficiency to some extent supported myogenic differentiation of RD cells.

### Dox-inducible c-Myb knockdown in RD xenografts is inefficient and tumor growth is not inhibited

**Figure 7:**
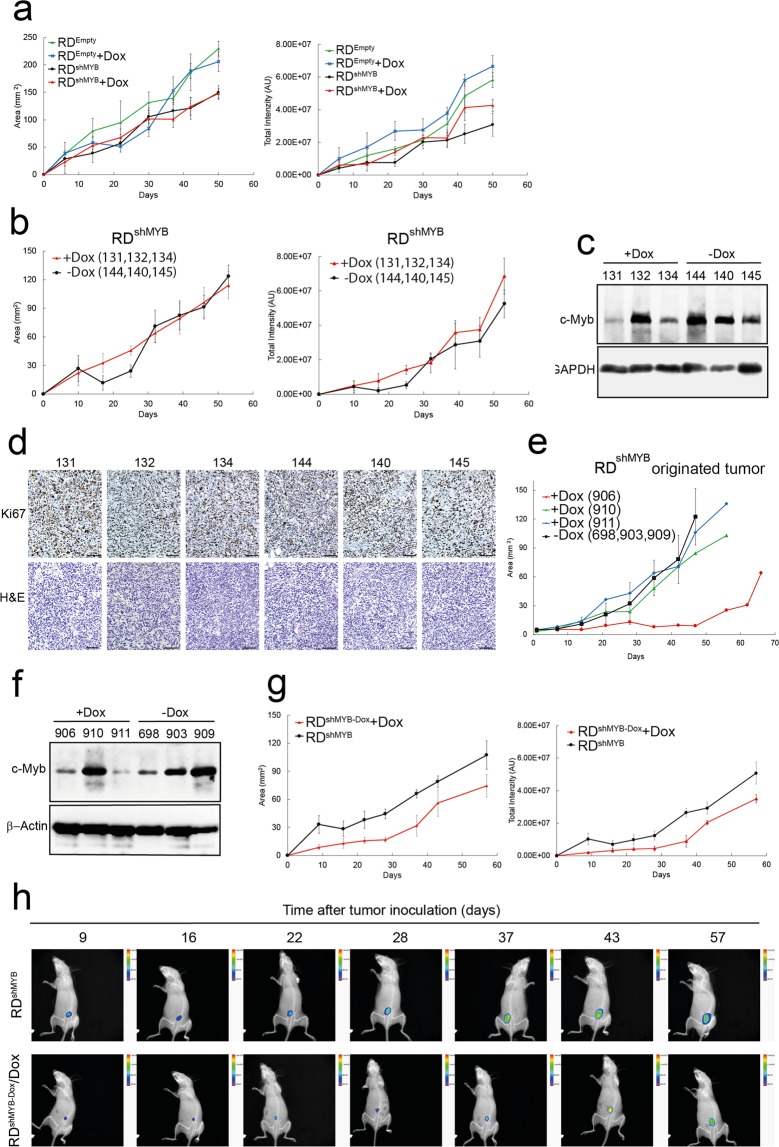
Analysis of RD xenografts. Analysed tumor xenografts in SCID mice were generated (**a–d**) using RD^shMYB^, control RD^Empty^ cells or (**g**,**h**) RD^shMYB-Dox^ cells that were treated with Dox for four days before harvesting and implantation (Day 0 on the graphs denotes inoculation of cells, Dox administration at day 7 or 10). (**e**,**f**) Alternatively, analysis of implanted RD^shMYB^ originated tumor specimens engrafted in NSG mice was done (Day 0 on graph denotes Dox administration). (**a**,**b**,**e**,**g**). Based on *in vivo* fluorescence, the tumor area or total emission was determined. (**a**,**g**) Dox was administrated in drinking water (2 mg/ml in 5% w/v sucrose) or (**b**,**e**) in the feed (625 mg/kg). (**c**,**f**) Western blot shows c-Myb expression in RD^shMYB^ cells generated tumors (**c**) or in tumors originated from xenotransplanted RD^shMYB^ tumors (**f**) excised from mice with or without Dox. GAPDH or β-Actin served as a loading control. The original full-length blots are presented in Supplementary Fig. 1. (**d**) Representative IHC images of H&E and Ki-67 staining for each tumor sample. Scale bars: 100 μm. (**h**) Time-course fluorescence imaging of RD xenograft tumors in mice. RD^shMYB^ or RD^shMYB-Dox^ cells were implanted and tumor growth was monitored (n = 4 mice per group in (**a**,**g**) n = 3 in (**b**,**e**).

After showing *in vitro* that c-Myb expression is an essential feature of eRMS tumorigenic cells (supporting proliferation, anchorage - independent growth, migration and invasiveness), we generated RD xenografts in SCID mice to investigate the role of c-Myb in RMS tumorigenesis *in vivo*. We used a Dox-inducible c-Myb shRNA to knockdown c-Myb expression. Since the cells constitutively express GFP, we uncovered arisen tumor by *in vivo* fluorescence imaging. Once the tumor was established and detected the drinking water was either supplemented with Dox (2 mg/ml in 5% w/v sucrose) (Fig. 7a), or a special diet (Dox 625 mg/kg) (Fig. 7b) was used. We evaluated tumor growth not only by quantification of the tumor area, but also by measuring total GFP signal from the cells forming the tumor tissue. Based on both tumor area and total GFP signal we did not observe any alteration in tumor growth after induction of c-Myb downregulation (Fig. 7a,b: red line). The tumors were also processed for imunohistological (IHC) analysis. IHC inspection surprisingly revealed that the c-Myb expression was not inhibited in tumors treated with Dox (Supplementary Fig. 7); the number of c-Myb-positive tumor cells was similar to the control tumor tissue. To obtain more precise quantification, we performed western blotting analysis for c-Myb (experimental set up: Dox in feed). We detected roughly similar c-Myb levels in all control tumors (Fig. 7c); nevertheless, in tumors treated with Dox in order to induce c-Myb silencing, the c-Myb levels greatly varied; in some tumor specimens the Dox treatment resulted in attenuation c-Myb expression. However, even the tumor with the lowest c-Myb level (tumor sample no.131) exhibited the same growth as a control tumor with high c-Myb expression (Fig. 7b). IHC analysis of Ki-67 staining of tumors (Fig. 7d) again confirmed that the proliferation rate of all investigated tumors was similar. We believe that the low levels of c-Myb found in tumor samples no. 131 and no. 134 are still high enough to support tumor growth similarly as the high levels of c-Myb found in control tumor samples.

Next, we performed serial xenotransplantation of tumor tissue originated from RD ^shMYB^ cell line (Dox treatment excluded) into NSG mice. Small portion of tumor were excised and inoculated into NSG mice. When tumor was detected by *in vivo* fluorescence imaging (intensity of GFP signal of engrafted tumor tissue immediately after xenotransplantation was too low to be detected), doxycycline was provided in feed (Dox 625 mg/kg). The delay of tumor growth was detected only in one of three Dox treated mice comparing to Dox untreated (Fig. 7e). Analysis of c-Myb expression in tumor samples was done by IHC and western blotting. IHC staining again revealed c-Myb expression in all tumor tissue samples, no matter if Dox was administered or not (not shown) and western blotting (Fig. 7f) analysis showed again great variation in c-Myb expression among Dox treated mice, from very low to high levels, similar to non treated mice. Dox treated mouse no. 906 exhibited delayed tumor growth and low c-Myb expression, however in none of analysed tumor samples c-Myb was ablated.

### c-Myb-deficient RD xenografts show poor tumor grafting efficiency and reduced tumor growth during initial stages of tumor expansion

We supposed that because c-Myb expression in RD xenografts was not efficiently suppressed, the effect of c-Myb on eRMS tumorigenesis was not observed. As we wished to generate RD xenografts with c-Myb suppressed, for implantation into SCID mice we therefore used c-Myb-deficient RD cells (Fig. 7g,h). RD^shMYB^ cells that were treated with Dox (RD^shMYB-Dox^). for four days in cell culture before harvesting and implantation into SCID mice. Once the RD^shMYB-Dox^ originated tumor was detected by *in vivo* fluorescence imaging the drinking water was supplemented with Dox (2 mg/ml in 5% w/v sucrose). The RD cells influenced by c-Myb knockdown already *in vitro*, in cell culture, before grafting into SCID mice, showed dramatic reduction in the graft propagation, suggesting that cells after exposure to c-Myb inhibition were less efficient in grafting into host tissues, which corresponds with the lower observed invasiveness *in vitro*. We also observed diminished tumor growth detectable up to 30 days after implantation of cells. However, later on, relative increments in tumor tissue mass growth did not show a significant difference from the control group. We believe that up to 30 days of tumor monitoring, the levels of c-Myb were kept low as a result of “cell culture suppression” of c-Myb but subsequently the original suppression was released, Dox induction was again ineffective to suppress c-Myb and tumors started to grow.

## Discussion

Our objective was to analyse whether c-Myb contributes to the RMS tumor phenotype, first, because c-Myb expression was identified in both aRMS and eRMS tumor specimens and shown to colocalize in tumor cell nuclei with myogenin, poor prognostic factor independent of the RMS histologic subtype^26^. The second reason was because RMS is a mesenchymal cancer associated with the skeletal muscle lineage^32^ and our results indicated that c-Myb inhibits skeletal muscle differentiation^25^.

The pLVTSH-Myb shRNA lentiviral vector^28^ expressing doxycycline (Dox)-inducible c-Myb shRNA was shown to effectively suppress c-Myb expression in *in vitro* assays^28,33–35^. We also achieved efficient knockdown of c-Myb expression in eRMS cells, resulting in inhibition of proliferation, colony formation, and migration, however, aRMS cells did not show any effect after c-Myb silencing and proliferated, migrated, and form colonies in soft agar at the same rate as control cells with high c-Myb levels. Moreover, we also documented, that upon c-Myb knockdown in eRMS cells, cells accumulated in G0/G1 phase and cell number in G2/M was reduced, the invasive behaviour of cells was repressed and elevated levels of myosin heavy chain, marker of muscle differentiation, was detected. As RH30^shMYB^ xenografts tumor growth equaled RD ^shMYB^ xenografts, we concluded that lentiviral transduction per se had no inhibitory effect on the ability of RH30 cells to form tumors *in vivo* (not shown). It is known that although both eRMS and aRMS are classified as two histological subtypes of RMS, they differ greatly. aRMS (RH30 as well) carry PAX3/7-FOXO1 fusion proteins (in more than 85% cases) that activate a set of transcription factors, but not c-Myb, driving RMS pathogenesis^36^. Because of that, c-Myb might be excluded from being involved in setting the cancer phenotype of aRMS. eRMS, on the other hand, do not carry such a driving mutation, and may arise from either satellite cells or myoblasts^32,37^. We have shown that c-Myb was expressed in activated satellite cells and descendants of activated satellite cells, proliferating myoblasts that were unable to fuse into myotubes when c-Myb was constitutively expressed^25^. As satellite cells and myoblasts are considered as potential cells of origin for embryonal rhabdomyosarcoma, we concluded, that in the process of conversion of myogenic progenitor cells into cancer cells, c-Myb, which originally regulated cellular differentiation, acquired oncogenic activity.

However, while the oncogenic activity of c-Myb was well documented *in vitro*, *in vivo* experiments using the RD xenograft model revealed that c-Myb efficient knockdown was not achieved and tumor growth was not inhibited. We observed reduction of c-Myb expression after Dox treatment, however we also demonstrated, that Dox treatment was ineffective in some cases, and the c-Myb levels were high, comparable to Dox untreated mice. We speculate that low c-Myb levels could still be high enough to support tumor growth as high c-Myb levels. To overcome this problem RD ^shMYB^ cells were subjected to Dox treatment for four days in cell culture, resulting in silencing of c-Myb expression before implantation into mice. Tumors originated from these cells exhibited diminished growth for period of 30 days and we suggest that c-Myb expression was silenced as a result of “cell culture suppression” of c-Myb. The same lentiviral vector expressing doxycycline (Dox)-inducible c-Myb shRNA has recently been used in *in vivo* study of c-Myb role in acute lymphoblastic leukemia^35^. The authors described delayed ineffective c-Myb suppression and reduction of GFP intensity in some Dox treated leukemic cells, suggesting that selection of leukemic cells expressing low levels of the MYB shRNA occurred. Our results, however, did not indicate reduced GFP intensity (signature of RD^shMYB^ cells) in tumor samples, in fact, GFP intensity was growing at the same speed as tumor area, suggesting that tumor tissue was propagated from authentic (GFP positive) RD^shMYB^ cells only. Therefore, to explain very different c-Myb expression levels after Dox induction, we suggest, that MYB shRNA levels under the control of Tet-repressor–regulated H1 promoter were reduced in some tumor specimens because of specific silencing of H1 promoter. Moreover, c-Myb levels, detected in tumor samples without Dox greatly exceeded levels detected in parental RD cell lines indicating that in growing tumors c-Myb expression increased. To explain such a variability in c-Myb expression in RD derived tumors we speculated that c-Myb expression could be subjected to intensive regulation in forming tumors, presumably reflecting site or implantation and tumor microenvironment.

To summarize, using lentiviral vector expressing Dox-inducible c-Myb shRNA in *in vitro* experiments resulted in efficient suppression of c-Myb and inhibition of eRMS tumorigenesis, however, in experimental tumors, Dox induction of Myb shRNA was less effective, and we detected various levels of c-Myb ranging from low levels to the levels similar to control tumor (detected by IHC and western blotting) with no inhibitory effect on tumor growth. We suggest that the low c-Myb levels presented in some experimental tumors supported growth similarly as the high c-Myb levels in control tumors. However, when RD xenografts were generated from already c-Myb-deficient RD cells, we observed inefficient tumor grafting and attenuation of growth during the initial stages of tumor expansion.

In conclusion, we suggest that c-Myb targeting can be an effective treatment strategy in eRMS providing ablation of c-Myb expression.

## Materials and Methods

### Cell lines and culture conditions

The C2C12 mouse myoblast cell line and RMS cell lines RD and RH30 were obtained from ATCC and cultured as recommended. To induce differentiation, 10% fetal bovine serum was replaced by 2% horse serum. For c-Myb suppression, the pLVTSH-Myb shRNA lentiviral vector (shMYB) and empty pLVTSH plasmid (Empty) were kindly provided by Dr.T. Gonda^28^. Lentiviruses were generated by cotransfection of HEK293T cells with packaging plasmids as described elsewhere. Supernatants were collected after 48 hours and used to transduce RD and RH30 cells. Transduced cells were FACS sorted on the basis of expression of pLVTSH-encoded GFP. Usually, transduced RMS cells were seeded, expression of c-Myb shRNA was induced next day by adding doxycycline (#D9891, Sigma) at a final concentration of 5 μg/ml and cells were analysed 48 hours later.

### Cell proliferation, cell cycle analysis and colony formation

To determine the number of viable cells in culture by measuring ATP present, cells were seeded at density 1.10^4^–1.10^5^/ml in a 96-well plate. Next day (Day 1), where indicated, cells were exposed to Dox. At treatment end points 100 μl of CellTiter-Glo® Luminescent Cell Viability Assay (#G7571, Promega) reagent was added and plates were analysed with a plate reader (Infinite 200 PRO, Tecan). To determine cell proliferation by crystal violet assay, cells were seeded in a 24-well plate and cultured as described above. At treatment day, cells were fixed with 4% paraformaldehyde in PBS for 15 min, washed with PBS, and stained with 0.1%crystal violet in water for 30 min. The stained cells were then washed with water, allowed to air dry over-night, the dye was eluted with 10% glacial acetic acid, and absorbance at 595 nm was measured. For cell-cycle analysis, cells were seeded at 2.10^4^, the next day, where indicated, the cells were exposed to Dox (final concentration 5 μg/ml) and grown for two or four days before cell-cycle analysis was performed by propidium iodide staining (20 μg/ml) of cells permeabilized with 0.1%TritonX-100 and treated with DNASe-free RNASe (# EN0531, Fermentas; 0.2 mg/ml) followed by flow cytometry determination of the DNA content. For the colony formation assay, cells were pretreated with Dox for 48 hours and seeded in 0.35% agar (Agar Noble, Difco) on top of 0.7% bottom agar containing Dox where indicated. Once a week the plates were overlaid with 1 ml medium containing Dox where indicated. Colonies were counted after 21 days. To induce myogenic differentiation, we cultured both myoblast cell line C2C12 and RD cells^38^ in low-serum medium (2% horse serum).

### Migration assay

Migration of cells was monitored by the scratch wound healing assay as described in^26^. Confluent cells pretreated with Dox for 48 hours, where indicated, were wounded with a comb and incubated for 72 hours to allow cells to migrate into the acellular area. To analyse single-cell suspension by time lapse microscopy, diluted cells seeded in a 24-well plate were pretreated for 48 hours with Dox and placed on a motorized stage of a Leica DMI 6000B microscope, and time-lapse microscopy for 48 hours was performed at one selected position of the stage (to prevent distortion of migration caused by movement of the stage) as described^25^. The trajectories of individual cells were obtained from fluorescence images (RD^shMYB^) and phase contrast images (RD) that were taken together every two minutes, with manual control and correction (see Supplementary Methods). The lateral movement of the cells was further analysed and characteristic measures were computed as staggered measures and represented as 2D heat maps. We calculated the staggered confinement ratio C_i_, the staggered displacement ratio D_i_, the staggered outreach ratio O_i_ and staggered volume asphericity A_d_ to characterize the tracks. The average staggered measures were computed and these values were compared between the two types of cells. Detailed description is given in the Supplementary Methods.

### Transwell assay

The transwell migration assay was performed using 8-μm pore size inserts (#351152, BD Falcon). Inserts were coated with 100 μl of Matrigel (#326231, Corning, diluted 30x in DMEM). Cells pretreated with Dox, where indicated, were plated at a density of 5.10^4^ cells/insert in the upper chambers and allowed to migrate for 48 hours, the bottom chambers contained the same growth medium. Cells on the upper side of the insert were removed with a cotton swab. The cells on the lower side of the insert were fixed with 4% paraformaldehyde, washed, stained by the DAPI, and counted.

### Western blotting, immunofluorescence, immunohistochemistry

Western blotting and immunofluorescence staining was performed as described previously^25^. Membranes were cut horizontally to allow for simultaneous detection of both investigated targets and loading controls, therefore, blots covering the entire range of molecular weights are not available. Cell extracts were analysed by monoclonal anti-Myb antibody (#05–175, clone1–1, Millipore) at dilution 1:250, by monoclonal anti-GAPDH antibody (#GTX30666, GeneTex) at dilution 1:2000, by monoclonal anti-β-Actin antibody (#A5316, Sigma-Aldrich) at dilution 1:5000, or monoclonal anti-myosin heavy chain (MHC) antibody (MF 20, Developmental Studies Hybridoma Bank) at dilution 1:400 according to the manufacturer’s instructions. For immunofluorescence staining MF20 was diluted 1:100. Immunohistochemistry was described in the Supplementary Methods.

### Mouse xenograft studies

For the xenograft study, 1.5.10^6^ RD^shMYB^, RD^Empty^, or RD^shMYB^ cells treated with Dox (5 μg/ml) for four days in cell culture before harvesting (RD^shMYB-Dox^), were diluted twice with Matrigel (#326231, Corning,) and implanted subcutaneously into the flanks of immunodeficient SCID/beige mice (Strain code 250 - Fox Chase SCID beige mice -CB17.Cg-PrkdcscidLystbg-J/Crl; Charles River). A week later, mice were anaesthetized with Zoletil® (50 mg/kg), shaven with Veet® Hair removal cream around the implantation site and subjected to *in vivo* imaging (fluorescence and X-ray) using *In-Vivo* Xtreme (Bruker). As soon as the tumor was detected (usually 7–10 days after implantation of cells), mice were randomly assigned to the experimental (+Dox) or control group (−Dox). In the experimental group, the drinking water was supplemented with 2 mg/ml doxycycline (#D9891, Sigma-Aldrich) in 5%w/v sucrose and replaced for fresh one every fourth day, or a complete feed with doxycycline (625 mg/kg, SSNIFF) was used. In the control group, the drinking water was supplemented with 5% w/v sucrose. Mice were monitored once a week. Mice were sacrificed upon reaching maximum tumor burden 2 000 mm^2^ (IACUC).

For xenotransplantation of RD^shMYB^ derived tumor in SCID mice (Dox was not administered) we used NSG mice (#00557, NOD.*Cg-Prkdc*^*scid*^*Il2rg*^*tm1Wjl*^/SzJ, The Jackson Laboratory). Part of a tumor was implanted into the recipient mice under aseptic conditions. The implantation was done after an incision on the back (5 mm) and the part of a tumor with size of 3 × 3 × 3 mm was implanted subcutaneously. Efficiency of xenotransplantation was 40%. We observed late tumor detection, comparing to RD cells, ranging from 1 to 3 weeks after implantation. The animals were handled in accordance with the Guide for the Care and Use of Laboratory Animals, approved by the Animal Care and Use Committee of the Academy of Sciences of the Czech Republic. Mice were kept under standard laboratory conditions with free access to food and water.

### Statistical analysis

Data shown represent one of three independent experiments, mean ± SD, n = 3. For analysis we used Student’s t-test (GraphPad Prism). P values were considered significant under 0.05, *P < 0.05, **P < 0.01, ***P < 0.001 vs Dox untreated.

## Supplementary information

Dataset 1

Supplementary Video
