# Supplementary material for: c-Myb regulates tumorigenic potential of embryonal rhabdomyosarcoma cells
Source: Sci Rep. 2019 Apr 19;9:6342. doi: 10.1038/s41598-019-42684-y (PMC6474878; doi:10.1038/s41598-019-42684-y)
Supplement: Supplementary file 1 — Dataset 1 [file 41598_2019_42684_MOESM1_ESM.docx]

c-Myb regulates tumorigenic potential of embryonal rhabdomyosarcoma cells

Petr Kaspar^1,^*, Jan Prochazka^1,3^, Michaela Efenberkova^2^, Attila Juhasz^3^, Vendula Novosadova^3^, Radislav Sedlacek^1,3,^*

^1^ Laboratory of Transgenic Models of Diseases, Institute of Molecular Genetics of the Czech Academy of Sciences, Prague, 142 20, Czech Republic

^2^ Microscopy Centre – LM and EM, Institute of Molecular Genetics of the Czech Academy of Sciences, Prague, 142 20, Czech Republic

^3^ Czech Centre for Phenogenomics Institute of Molecular Genetics of the Czech Academy of Sciences, Prague, 142 20, Czech Republic

* Corresponding authors

Correspondence to Radislav Sedlacek or Petr Kaspar

Radislav Sedlacek:

Laboratory of Transgenic Models of Diseases, Institute of Molecular Genetics of the Czech Academy of Sciences Vídeňská 1083, Prague 4, 142 20, Czech Republic

E-mail: [radislav.sedlacek@img.cas.cz](mailto:radislav.sedlacek@img.cas.cz) | Phone: (+420) 325 873 243-2 | Fax: (+420) 224 310 955

Petr Kaspar:

Laboratory of Transgenic Models of Diseases, Institute of Molecular Genetics of the Czech Academy of Sciences, Vídeňská 1083, Prague 4, 142 20, Czech Republic

E-mail:petr.kaspar@img.cas.cz | Phone: (+420) 325 873 249

SUPPLEMENTARY INFORMATION

**Supplementary Methods**

Immunohistochemistry

Tumors were fixed in 4% formaldehyde in 1xPBS for 48 hours. After the fixation they were transferred into 70% ethanol and continuously processed automatically by a Leica 420 Tissue Processor. Dehydration and paraffine fulfillment were executed according to a 12 hour standardized protocol. The tissue samples were then embedded into FFPE blocks and cut into SuperFrost+ slides at 3 μm of thickness. After deparaffination and washing in 1xPBS, heat-induced antigen retrieval was provided at pH 6 citrate buffer 1x (Zytomed HIER pH6). After cooling down, the slides were washed in 1x PBS for 2x5 min. at RT. Blocking of endogenous peroxidase activity was performed for 20 min at RT in 3% hydrogen peroxide in methanol. After washing in 1x PBS for 2x5 min at RT, blocking of non-specific binding sites was provided with 2% BSA (#0163.4, Carl Roth GMBH) in 1x PBS for 20 min at RT. c-Myb staining was done using rabbit polyclonal c-Myb antibody (#LS–B5315, LSBio) at dilution 1:1500 in Zytomed Antibody Diluent, with incubation at 4°C o/n, and the slides were then washed in Zytomed Buffer 1x for 2x5 min at RT. Biotinylated secondary antibody used was anti-rabbit HRP-conjugated 1:500 diluted in 2% BSA, with incubation at RT for 30 min. Signal amplification was provided with ABC System (#PK-6100, Vectastain Elite ABC Kit - HRP Conjugated). The slides were incubated for 30 min at RT. Visualisation was done with DAB. Ki-67 antibody was diluted 1:500 (#MA5-14520, Thermofisher).

Cell track analysis

Time-dependent positions of individual cells were obtained from fluorescence images of RD^shMYB^ and phase contrast images of RD cells, respectively. Individual cell positions were recorded in FiJi after correction of the background and contrast adjustment. We decided to use the TrackMate plugin (1) to record the lateral positions of the centers of the cell nuclei in time; cell nuclei represented the structures that were well distinguishable in the phase contrast images. Selected cell trajectories were manually controlled and corrected. The lateral movement of the cells was further analyzed only for cells with the total track length at least 1000 minutes, i.e. 500 time points. Due to the long duration of the tracks the cells overlapped in some parts, which made the localization of individual cells in these time points impossible. These missing parts of the trajectories were not taken into account in the analysis. At least 30 cells were analyzed in each case. All computations were performed in Matlab software (Release 2015a, The MathWorks, Inc., Natick, MA, USA).

Characteristic measures were computed as staggered measures according to (2) as a function of time for varying starting time points along the track, in contrast to linear measures that are computed along the track of a cell relative to its initial position only. The resulting characteristics depend on all possible combinations of a cell track’s time-ordered segments and are represented as 2D heat maps.

The staggered confinement ratio of i-th cell track is an extension of the confinement ratio determined by the displacement vector between time points m and n+1, where m≤n

$$\vec{d}_{i}\left( n,m \right)=\vec{r}_{i}\left( n+1 \right)-\vec{r}_{i}(m)$$

over the length of the corresponding cell track segment l_i_, defined as total track length between time points m and n+1. The entries of confinement ratio matrix C_i_ are then represented by

$$C_{i}\left( n,m \right)=\frac{\left| \vec{d}_{i}(n,m) \right|}{l_{i}(n,m)}.$$

The staggered displacement ratio is defined similarly to the staggered confinement ratio, where instead of length of the cell track the longest displacement vector d_i_^max^(n,m) between time points m and n+1 is used. The staggered displacement ratio D_i_ is then represented by

$$D_{i}\left( n,m \right)=\frac{\left| \vec{d}_{i}(n,m) \right|}{d_{i}^{max}(n,m)}.$$

The entries of the staggered outreach ratio O_i_ are defined as staggered confinement ratio over staggered displacement ratio

$$O_{i}\left( n,m \right)=\frac{C_{i}(n,m)}{D_{i}(n,m)}=\frac{d_{i}^{max}(n,m)}{l_{i}(n,m)},$$

i.e. the maximal displacement length over the length of the track segment.

The shape of the track volume is characterized by staggered volume asphericity, A_d_ which quantifies the deviation of the track volume from a d-dimensional sphere, d=2 in this case. It is given by gyration radii R_1_ and R_2_ from the gyration tensor that defines the ellipsoidal volume covering the data points from the cell track, for i-th cell track

$$A_{i}\left( n,m \right)=\frac{{(R_{1}^{2}-R_{2}^{2})}^{2}}{{(R_{1}^{2}+R_{2}^{2})}^{2}}.$$

An average staggered value is subsequently computed for each cell track. The linear measures (1D time-dependent curves) for individual cells correspond to the first column of the staggered confinement ratio C_i_(n,1), staggered displacement ratio D_i_(n,1), staggered outreach ratio O_i_(n,1) and staggered volume asphericity A_i_(n,1), respectively. Thus, the staggered measures are more sensitive to the local migration behavior in comparison with the linear measures and similarly, the average values calculated from the staggered measures yield more precise results when compared to the linear measures.

1. Tinevez JY, Perry N, Schindelin J, Hoopes GM, Reynolds GD, Laplantine E, et al. TrackMate: An open and extensible platform for single-particle tracking. Methods. 2017;115:80-90.

2. Mokhtari Z, Mech F, Zitzmann C, Hasenberg M, Gunzer M, Figge MT. Automated characterization and parameter-free classification of cell tracks based on local migration behavior. PLoS One. 2013;8(12):e80808.

Primers

Primers used for qPCR:

MYB forward: AAACAAGGTGGACCCGGGAAG,

reverse: ACTGCACATCTGTTCGATTCGG

GAPDH forward: CCCATCACCATCTTCCAGGAG,

reverse: CTTCTCCATGGTGGTGAAGACG

**Supplementary Figures and Video Legend**

**Supplementary Figure 1**

**Full-length blots of Figure 1a**

The red marks indicate bands showed in Figure 1a

**
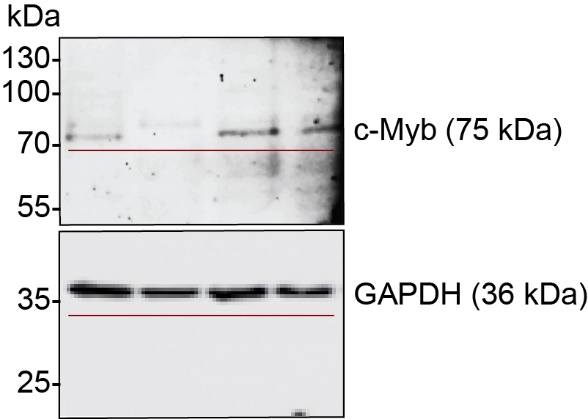
**

**Full-length blots of Figure 1e**

The red marks indicate bands showed in Figure 1e


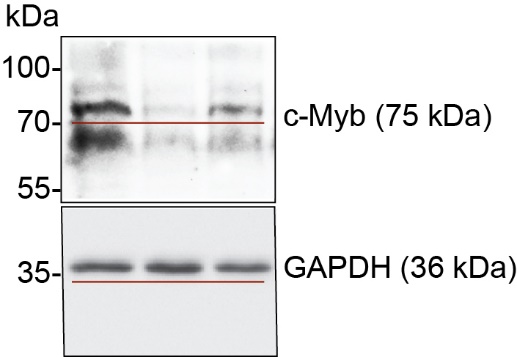


**Full-length blots of Figure 6**

The red marks indicate bands showed in Figure 6

**
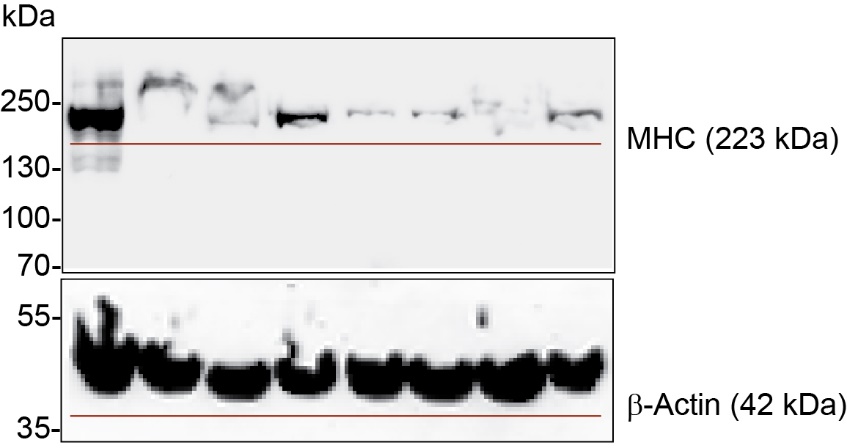
**

**Full-length blots of Figure 7c**

The red marks indicate bands showed in Figure 7c

**
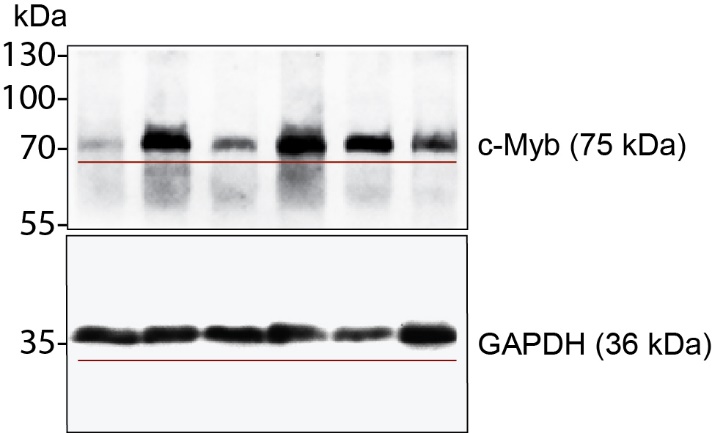
**

**Full-length blots of Figure 7f**

The red marks indicate bands showed in Figure 7f

**
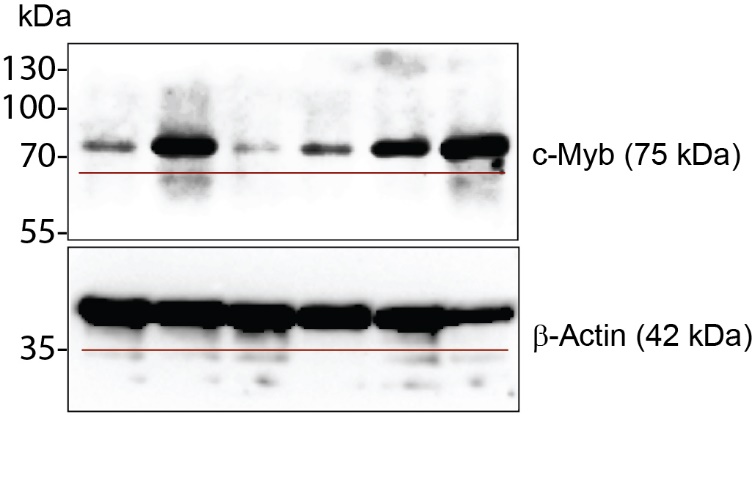
**

**Supplementary Figure 2**

**
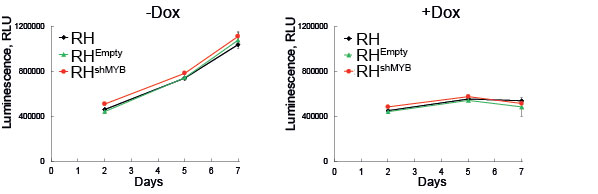
**

**Supplementary Figure 2.Sensitivity of RH30 cells to Dox at 5 μg/ml.** Cells were grown with or without Dox (5 μg/ml), as indicated, and proliferation rate was measured by ATP assay.

**Supplementary Figure 3**

**
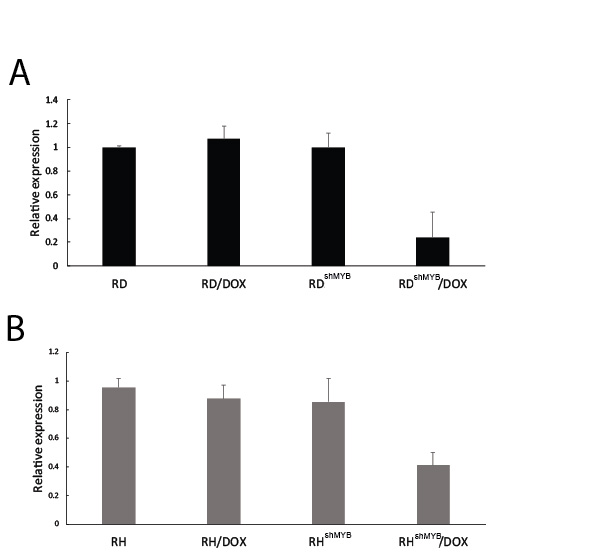
**

**Supplementary Figure 3.c-Myb mRNA levels determined by qPCR.** Cells were grown with or without Dox (5 μg/ml for RD, 2.5 μg/ml for RH30) for 48 hours.

**Supplementary Figure 4**

**
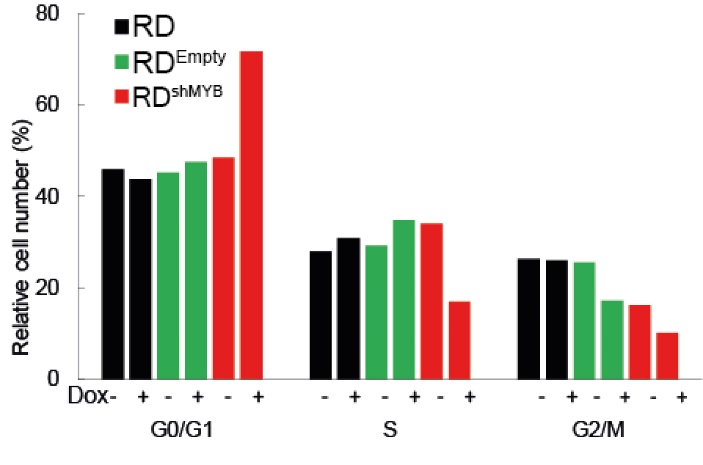
**

**Supplementary Figure 4. Knockdown of c-Myb in RD for two days block cell cycle progression.** Cells were grown with or without Dox (5 μg/ml), as indicated, for two days and analysed by propidium staining and flow cytometry (data shown represent one of three independent experiments mean +/-SD, n=3, *p<0.01*).

**Supplementary Figure 5**


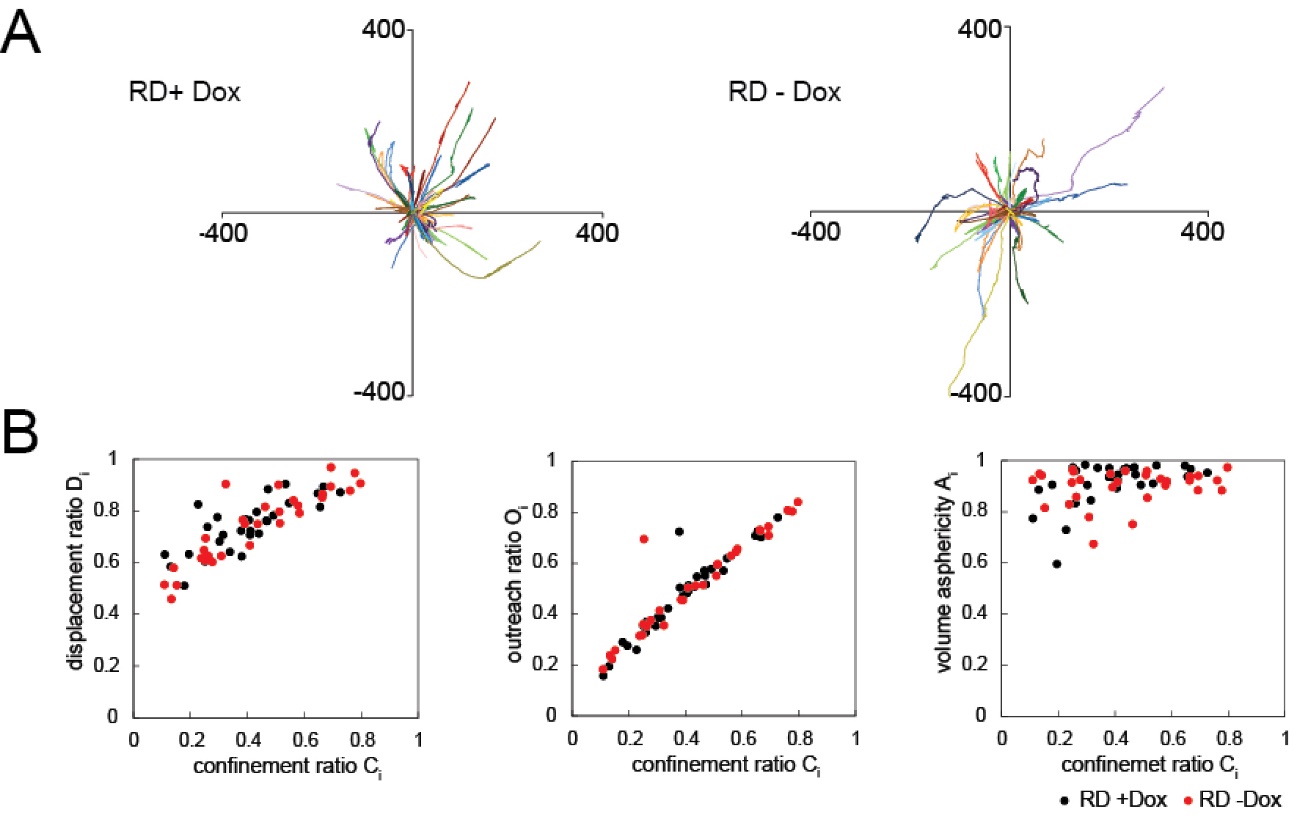


**Supplementary Figure 5. Dox treatment does not influence migration of parental RD cells.** Analyses of cell track data for RD cells with and without Dox. (**A**) Plot of individual RD cell tracks after alignment of starting positions. Number of cell tracks *n_+Dox_ = 30*, *n_-Dox_ = 30*. (**B**) Individual cell tracks depicted in the space defined by the average confinement ratio versus average displacement ratio, average outreach ratio, and average volume asphericity, respectively. Wilcoxon rank-sum test was used to compare the average staggered measures of +Dox and –Dox cells. There was no significant difference among any of the measures between RD with or without Dox (*p>0.05).*

**Supplementary Figure 6**

*
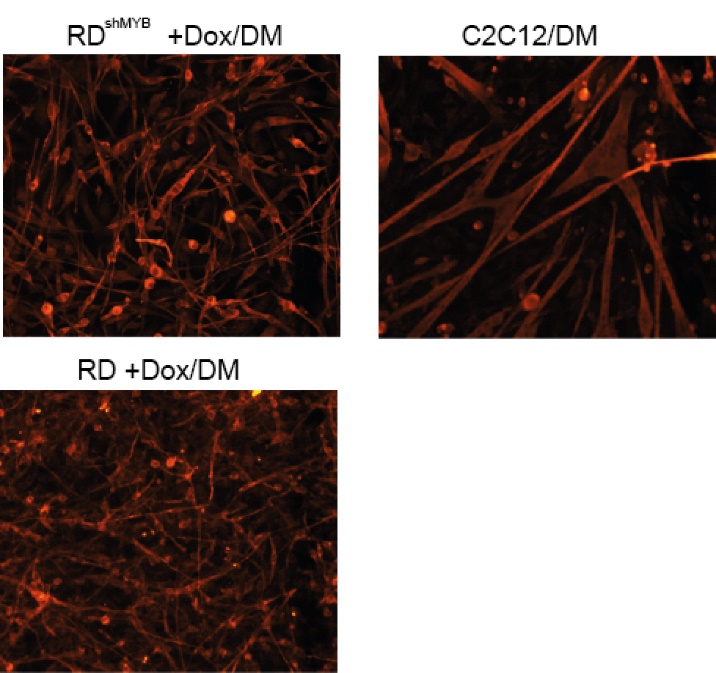
*

**Supplementary Figure 6. c-Myb silencing does not promote cell fusion in differentiating eRMS cells.** Cells were cultured in differentiation medium (DM) with or without Dox (5 μg/ml), and stained for marker of skeletal muscle differentiation MHC. Despite the massive fusion seen in C2C12 myoblasts, c-Myb-deficient RD cells did not fuse and morphologically resembled parental RD cells.

**Supplementary Figure 7**


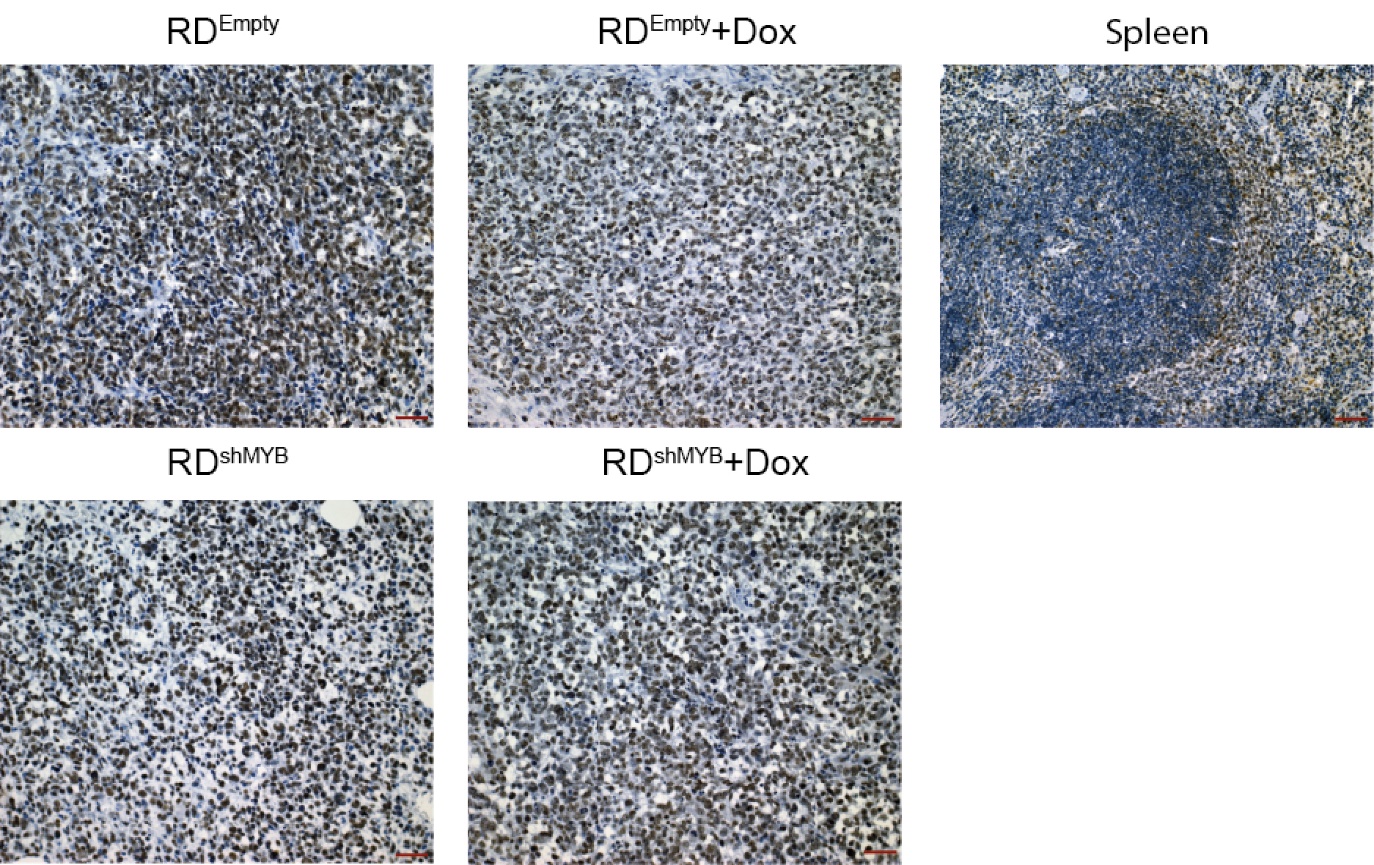


**Supplementary Figure 7. Immunohistochemical staining for c-Myb in RD xenografts tumor samples.** Representative IHC images of c-Myb staining. Spleen served as a positive control (the Human Protein Atlas: www.proteinatlas.org) Scale bars: 50 μm.

**Supplementary Video Presentation**

Time-lapse imaging of RD^shMYB^ and RD cells. The trajectories of individual cells were obtained from fluorescence images (RD^shMYB^) and phase contrast images (RD) that were taken together every two minutes. Cell were monitored for 48 hours. The frame rate is 18 fps, frame size is 546 x 437 pixels (original frame size is 1392 x 1040), pixel size 0.9170575 μm.
